# Serum Metabolomics in PCOS Women with Different Body Mass Index

**DOI:** 10.3390/jcm10132811

**Published:** 2021-06-25

**Authors:** Katarzyna Ożegowska, Szymon Plewa, Urszula Mantaj, Leszek Pawelczyk, Jan Matysiak

**Affiliations:** 1 Department of Infertility and Reproductive Endocrinology, Poznan University of Medical Sciences, 61-701 Poznań, Poland; leszek.a.pawelczyk@gmail.com; 2 Department of Inorganic and Analytical Chemistry, Poznan University of Medical Sciences, 61-701 Poznań, Poland; szymonplewa1@gmail.com (S.P.); jmatysiak@ump.edu.pl (J.M.); 3 Division of Reproduction, Medical Faculty I, Poznan University of Medical Sciences, 61-701 Poznan, Poland; urszula.mantaj@gmail.com

**Keywords:** polycystic ovary syndrome, metabolome, metabolic disturbances, body mass index, insulin resistance, metabolic pathways

## Abstract

Polycystic ovary syndrome (PCOS) is the most prevalent endocrine and metabolic disorder, affecting 5–10% of women of reproductive age. It results from complex environmental factors, genetic predisposition, hyperinsulinemia, hormonal imbalance, neuroendocrine abnormalities, chronic inflammation, and autoimmune disorders. PCOS impacts menstrual regularities, fertility, and dermatological complications, and may induce metabolic disturbances, diabetes, and coronary heart disease. Comprehensive metabolic profiling of patients with PCOS may be a big step in understanding and treating the disease. The study aimed to search for potential differences in metabolites concentrations among women with PCOS according to different body mass index (BMI) in comparison to healthy controls. We used broad-spectrum targeted metabolomics to evaluate metabolites’ serum concentrations in PCOS patients and compared them with healthy controls. The measurements were performed using high-performance liquid chromatography coupled with the triple quadrupole tandem mass spectrometry technique, which has highly selective multiple reaction monitoring modes. The main differences were found in glycerophospholipid concentrations, with no specific tendency to up-or down-regulation. Insulin resistance and elevated body weight influence acylcarnitine C2 levels more than PCOS itself. Sphingomyelin (SM) C18:1 should be more intensively observed and examined in future studies and maybe serve as one of the PCOS biomarkers. No significant correlations were observed between anthropometric and hormonal parameters and metabolome results.

## 1. Introduction

Polycystic ovary syndrome (PCOS) is one of the most prevalent endocrine and metabolic disorders. It results, i.e., from complex and poorly understood environment factors, genetic predisposition, hyperinsulinemia, hormonal imbalance, neuroendocrine abnormalities, chronic inflammation, and autoimmune disorders [1,2,3]. However, it is mainly characterized by clinical and biochemical hyperandrogenism, ovulatory dysfunction, and the polycystic morphology of the ovaries on the ultrasound [4]. Therefore, various classifications are proposed to diagnose PCOS: National Institutes of Health Criteria (NIH), defined in 1990, include the presence of clinical and/or biochemical hyperandrogenism and oligo/amenorrhea anovulation [5].

In 2003 the European Society of Human Reproduction and Embryology/American Society for Reproductive Medicine Rotterdam consensus (ESHRE/ASRM) evolved NIH criteria, requiring two of three features: clinical and/or biochemical hyperandrogenism, anovulation or oligo-ovulation, and polycystic ovarian morphology (PCOM) on the ultrasound [6]. The Rotterdam criteria are controversial since fulfilling two of three diagnostic criteria implies that PCOS can be diagnosed in the absence of androgen excess or menstrual irregularity [7]. In 2006, the Androgen Excess Society (AES) reviewed existing data on phenotypic expression. They concluded that there was conflicting evidence supporting the presence of such features as insulin resistance and metabolic disturbances in women with polycystic ovaries and ovulatory dysfunction, but without clinical or biochemical signs of hyperandrogenism. Thus, the AES considered that androgen excess is a central feature in the development and pathogenesis of polycystic ovary syndrome and proposed the definition that androgen excess should be present and accompanied by oligomenorrhea or PCOM or both of them. In addition to those features, exclusion of other androgen excess causes (i.e., non-classical congenital adrenal hyperplasia (NC-CAH), Cushing’s syndrome, androgen-secreting tumors, hyperprolactinemia, thyroid diseases, drug-induced androgen excess, disorders) is essential for the diagnosis [8]. It affects 5–21% of women of reproductive age [1,9], depending on the different geographic regions, as well as the criteria used to diagnose the syndrome: 5% to 10% according to NIH 1990 criteria; 10% to 15% according to the AE-PCOS 2006 criteria, and 6% to 21% by ESHRE/ASRM 2003 criteria [10,11,12,13,14,15].

In 2018, Teede et al. proposed an international evidence-based guideline for the assessment and management of PCOS that encourages Rotterdam criteria in diagnosing PCOS [16].

Insulin resistance (IR) and hyperandrogenemia are deemed to be the most basic pathophysiological changes in PCOS. In addition, PCOS impacts menstrual regularities, fertility, and dermatological complications and may induce metabolic disturbances, diabetes, and coronary heart disease [17,18].

A vast group of PCOS patients is overweight or obese, leading to abdominal and visceral obesity [19,20]. Independently of obesity, those women often (50–70%) present with IR and compensatory hyperinsulinemia [19,21]. Researchers have attempted to determine whether biomarkers might use as predictive markers for different phenotypes.

In 2012, the NIH consensus panel proposed to classify PCOS according to phenotypes. Phenotype A (full-blown syndrome PCOS: clinical or biochemical hyperandrogenism (HA)+ ovulatory dysfunction (OD)+polycystic ovarian morphology (PCOM) (HA + OD + PCO). Phenotype B (non-PCO PCOS: HA + OD) includes hyperandrogenism (HA) and ovulatory dysfunction (OD). Phenotype C (ovulatory PCOS: HA + PCOM) includes hyperandrogenism (HA) and polycystic ovaries (PCOM). Phenotype D (non-hyperandrogenic PCOS: OD + PCOM) includes ovulatory dysfunction (OD) and polycystic ovaries (PCOM) [22].

Homeostatic model assessment of insulin resistance (HOMA-IR) [23], the testosterone-to-androstenedione ratio [24], free androgen index [25], serum anti-mullerian hormone (AMH) level [26] was found to be potential biomarkers for predicting and diagnosing PCOS. Thus, it is essential to understand the disease’s pathogenesis better and identify its potential biomarkers, which may help us diagnose it correctly, seek prevention agents, and effectively manage PCOS symptoms and complications. Comprehensive metabolic profiling of patients with PCOS may be a big step in understanding and treating the disease.

Metabolomics is a rapidly advancing field of discovery science aimed at furthering knowledge of the biological consequences of metabolic changes from a single cell to the whole organism [27]. As a method of analysis, it allows the investigation of underlying mechanisms that control biological functions and the expression of different phenotypes of patients [28]. Combined with genomic and proteomic data, it might show both the organism’s physiological and pathological state [29]. In metabolomics, there are two approaches: untargeted, which usually analyzes the numbers of metabolites in the samples without a priori knowledge about these compounds, and the second-targeted. The targeted metabolomics is typically used for qualitative and quantitative analysis of selected metabolites or metabolites. Today it is possible to analyze even over the hundred metabolites from different chemical classes using a targeted approach. Such an approach might be helpful to identify and quantify multiple small-molecule metabolites such as amino acids, lipids, nucleotides, and organic acids in biological samples. Compared to proteomics, transcriptomics, and genomics, it is considered closer to the actual phenotype and gives us the most helpful information that may serve as a utensil for personalized diagnosis, treatment, and disease monitoring [30,31]. The analysis of metabolic differences between patients and healthy controls can indicate potential biomarkers of the disease, lead to a more proper understanding of its pathogenesis, and help control and evaluate the diagnostic and treatment methods [32]. Metabolites can be intrinsic, resulting from normal cellular physiology, or extrinsic, with the influence of exogenously administered drugs or interventions [33]. The Human Metabolome Database (HMDB) contains up to May 2020 114,186 metabolite entries, including both water-soluble and lipid-soluble metabolites, as well as metabolites that would be regarded as either abundant (>1 μM) or relatively rare (<1 nM). Additionally, 5702 protein sequences are linked to these metabolite entries.

Because PCOS is a complex disorder, complicated with androgen excess, abdominal obesity, IR, and various metabolic disturbances, which leads to a different manifestation of the disease and presentation with different phenotypes [34], there is a need to examine the exact origin of these metabolic alterations and therefore to seek for new metabolic biomarkers for PCOS [35]. The complex metabolic pathways involved in the occurrence of PCOS and its symptoms are not well understood. Some studies evaluated carbohydrate, lipid, and protein metabolism in this group of patients [36,37,38].

The presented study aimed to evaluate metabolites in average weight and obese patients with PCOS diagnosis and compare the results with a healthy population, using the well-established high-throughput Liquid Chromatography–Mass Spectrometry (LC-MS/MS) methodology with a proven interlaboratory reproducibility.

## 2. Materials and Methods

### 2.1. Study Subjects

We included into the study patients diagnosed with PCOS according to Rotterdam criteria [8]: (1) the presence of clinical and/or biochemical signs of hyperandrogenism (Ferriman-Gallwey score ≥8; testosterone level >0.5 ng/mL); (2) oligo- or anovulation (<8 cycles/year) and/or polycystic ovaries morphology on the ultrasound (ovarian volume> than 10 mL provided excellent specificity for PCOS in a majority of studies and used 12 or more follicles of 2 to 9 mm) [39]. In all the patients, congenital adrenal hyperplasia was excluded by evaluating morning follicular phase 17-hydroxyprogesterone (<2 ng/mL), hyperprolactinemia was excluded by morning prolactin levels. Cushing’s syndrome and androgen-secreting tumors were excluded based on clinical evaluation.

PCOS patients were divided into two groups according to BMI. According to the World Health Organization (WHO), average body weight is considered BMI ≥ 18.5 to 24.9 kg/m^2^. As abnormal BMI, WHO considers overweight patients with BMI ≥ 25 to 29.9 kg/m^2^ and obesity with BMI ≥ 30 kg/m^2^ [40].

We divided the study group into abnormal bodyweight group (PCOS-1) with 34 patients with diagnosed PCOS and BMI ≥ 25 kg/m^2^ and PCOS-2 with 32 women with PCOS and normal BMI (<25 kg/m^2^).

We matched those two groups with age-matched healthy controls (*n* = 11), which consisted of average women who came for a routine check-up in our hospital’s outpatients’ clinic. All the patients we recruited at the Gynecological and Obstetrical Hospital of Poznan University of Medical Sciences from 2018 to 2019.

The control group consisted of healthy volunteers with regular menstrual cycles, no clinical or biochemical hyperandrogenism, routine ovarian ultrasonography, no history of endocrine or autoimmune disorders, and no pelvic history surgery. In all the groups, we excluded patients with:

- Neoplasm
- Kidney failure
- Hypertension, dyslipidemia, diabetes mellitus
- Other endocrinopathies
- Hormonal treatment
- Drugs influencing the glycemic levels

### 2.2. Patients Evaluation

Medical and family history we investigated for all patients. In addition, clinical examination was performed, including measurement of body weight, height, waist circumference (WC) at the midpoint between the lateral iliac crest and the lowest rib margin at the end of normal expiration, waist to hip ratio (WHR), and hip circumference (HC) measured at the broadest level of the greater trochanters. Body mass index (BMI) was calculated as weight in kilograms divided by the square of height in meters (kg/m^2^). According to the World Health Organization categories, being overweight was defined as having a BMI of 25.0–29.9 kg/m^2^, and obesity was described as a BMI of ≥30.0 kg/m^2^ [40]. All the patients enrolled in the study were evaluated during the menstrual cycle’s early follicular phase (days 3–5) after discontinuing antidiabetic and contraceptive agents for ≥three months.

## 3. Methodology

### 3.1. Biochemical and Hormonal Analysis

Blood samples for biochemical and hormonal analysis were drawn from the antecubital vein between 8 and 10 AM following a 12-h overnight fast. The serum/plasma specimens were stored at −70 °C. The 75 g oral glucose tolerance test was performed in all patients; blood samples were obtained at baseline and at 1 and 2 h postprandially to determine glucose and insulin at each time point. Serum concentrations of estradiol (E2), luteinizing hormone (LH), follicle-stimulating hormone (FSH), prolactin (PRL), total testosterone (T), dehydroepiandrostendion sulfate (DHEA-S), sex-hormone-binding globulin (SHBG), thyroid-stimulating hormone TSH, and thyroxine (fT4) were measured by immunoenzymatic assay (ELISA Kit, Invitrogen, Thermo Fisher Scientific, USA). All biochemical, hormonal and standard coagulation panel analyses were performed in the university hospital’s accredited laboratory, holding certificates of quality management ISO 9000.

### 3.2. Metabolome

We collected serum samples into 7.5 mL S-Monovette (Sarstedt AG&Co., Nümbrecht, Germany) tubes with a clotting activator to evaluate metabolome. After 30 min of room temperature storage, samples were centrifuged (15 min. at 4000 rpm), serum was aliquoted, frozen, and stored at −80 °C. Before analysis, the serum samples were thawed and centrifuged at 2800× *g* for 5 min.

The AbsoluteIDQ p180 kit (Biocrates Life Sciences AG, Innsbruck, Austria) was used to evaluate the broad spectrum of metabolites in samples. This high-throughput methodology with a proven interlaboratory reproducibility [41] enables the analysis of up to 188 metabolites from a different class of chemical compounds. The analyses of amino acids and biogenic amines required chromatographic separation accomplished by LC-MS/MS experiment. In turn, the analysis of acylcarnitine, hexoses, and lipids was accomplished by the flow injection analysis (FIA-MS/MS) experiment performed after the LC-MS/MS experiment.

The sample preparation procedure was performed step-by-step by the manufacturer’s specifications. Firstly, calibration standards, quality control samples, and internal standards mix dissolved inappropriate water and shook to mix. Next, the 10 µL of the standard internal mix was pipetted to each well of the 96-well plate, followed by pipetting 10 µL of serum–sample and drying the plate under nitrogen flow. Afterwards, 50 μL of 5% phenylisothiocyanate was added, and the plate was incubated for 20 min, after which drying for 60 min under nitrogen was applied. Then, extraction with 300 µL of 5 mM methanolic solution of ammonium acetate was carried out. The extraction was performed by shaking the plate with extraction solvent for 30 min at 450 rpm, and then nitrogen flow was used for transferring the extract to the capture plate. In the end, the extract was split and diluted with appropriate solvents, giving two separate plates, first for LC-MS/MS experiment, the second plate for the FIA experiment.

The 1260 Infinity high-performance liquid chromatography (Agilent Technologies, Santa Clara, CA, USA) coupled to a triple quadrupole tandem mass spectrometer (SCIEX, Framingham, MA, USA) was used for metabolite quantitation. Chromatographic separation was achieved by application the ZORBAX Eclipse XDB-C18 (3.0 × 100 mm, 3.5 µm) column (Agilent Technologies, Santa Clara, CA, USA), with a pre-column (C18, 4.0 × 3.0 mm) SecurityGuard (Phenomenex, Torrance, CA, USA). The liquid chromatography and mass spectrometer were operated, and the data was acquired under the control of Analyst software version 1.6 (Sciex, Framingham, MA, USA). Biocrates MetIDQ software (Biocrates Life Sciences AG, Innsbruck, Austria) was used for data processing.

### 3.3. Statistical Analysis

All statistical analyses were performed using the Statistica version 10 PL software (StatSoft, Inc., Tulsa, OK, USA). The distribution of continuous variables was evaluated using the Shapiro–Wilk test. Because of the absence of normal distribution, nonparametric testing was performed using Mann-Whitney U and Kruskal–Wallis tests. Continuous variables were expressed as medians (interquartile range (IQR), 25–75th percentile) unless otherwise indicated. *p* < 0.05 was considered a statistically significant difference between medians.

In statistical testing of determined metabolites, unsupervised multivariate principal component analysis (PCA) was used to find potential outliers and examine clustering or separation trends. In addition, for comparing patients with PCOS and the control group, the volcano plot analysis was carried out. For this analysis, which is a combination of fold change and t-tests, the following thresholds were set: 1.25 and 0.05 for fold change and *p*-value, respectively. Moreover, univariate ROC curve analysis was performed for searching for the highest discriminating potential of determining compounds. Additionally, the correlation analysis between metabolome results and clinical parameters was carried out. Finally, the Metaboanalyst web portal was applied for targeted metabolomics data analysis and visualization [42].

The Ethics Committee approved the study protocol of Poznań University of Medical Sciences (Poznan, Poland). Approval number 741/20 (4.11.2020). Written consent was obtained from all the subjects.

## 4. Results

In Table 1, we present characteristics of the whole PCOS group compared with controls. Patients were age-matched with a median age of 28.5 (28.0–32.0) in PCOS patients and 26 (24.7–30.5) in the control group. We see a significant difference in the patients’ anthropometric parameters (BMI, WC, HC, WHR) and metabolic parameters (fasting glucose and insulin levels, HOMA-IR, complete lipid profile).

When we divided PCOS patients into groups according to BMI level and compared them with a control group, we notice significant differences in similar parameters (Table 2). Patients did not differ according to age, but we may detect a substantial elevation of BMI, WC, HC, and WHR in PCOS-1 than PCOS-2 and controls. In addition, the metabolic profile (glucose levels, insulin levels, HOMA-IR, HbA1%C) was significantly different between PCOS groups than in controls. PCOS-Obese patients also had the most abnormal lipid profile. Interestingly PCOS-2 patients and controls did not differ at all following C-reactive protein (CRP), but it was significantly higher in the PCOS-obese population.

In Table 3 we present the hormonal analysis of the three groups. We may notice the elevation of testosterone (T) levels in both PCOS groups (median >0.5 ng/mL) as well as typical for hyperandrogenism drop in the level of sex-hormone globulin (SHBG) in PCOS populations.

### 4.1. PCA

**Figure 1:**
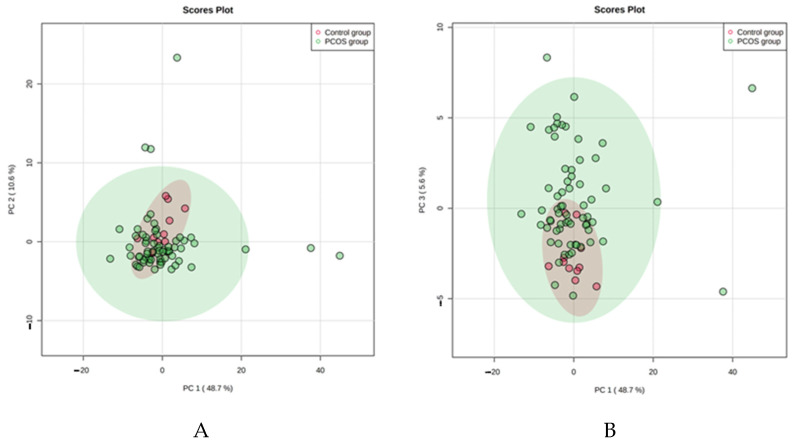
The unsupervised multivariate principal component analysis (PCA) of the studied metabolites in the PCOS and control groups. The plots represent the first principal component (PC1) against the second principal component (PC2) (**B**) and the first principal component (PC1) against the third principal component (PC3) (**A**).

We used the unsupervised multivariate principal component analysis (PCA) to examine clustering or separation trends and find potential outliers. Figure 1 showed a separation tendency among data from the control group and the whole PCOS population. We may observe on this figure some partial separation of samples (outliners) in the PCOS group that differ significantly from the rest of the group. Interestingly, BMI, age, hormonal and lipid profile, anthropometric parameters did not vary between those outliners and the rest of the PCOS group and therefore probably had no impact on sample clustering.

The unsupervised multivariate principal component analysis (PCA) of the studied metabolites in the PCOS and control groups.

**Figure 2:**
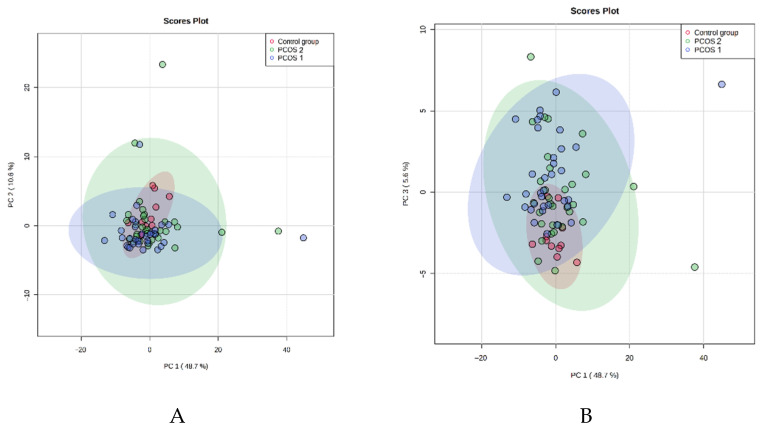
The unsupervised multivariate principal component analysis (PCA) of the studied metabolites in the PCOS-1, PCOS-2, and control groups. The plots represent the first principal component (PC1) against the second principal component (PC2) (**A**) and the first principal component (PC1) against the third principal component (PC3) (**B**).

**Figure 3:**
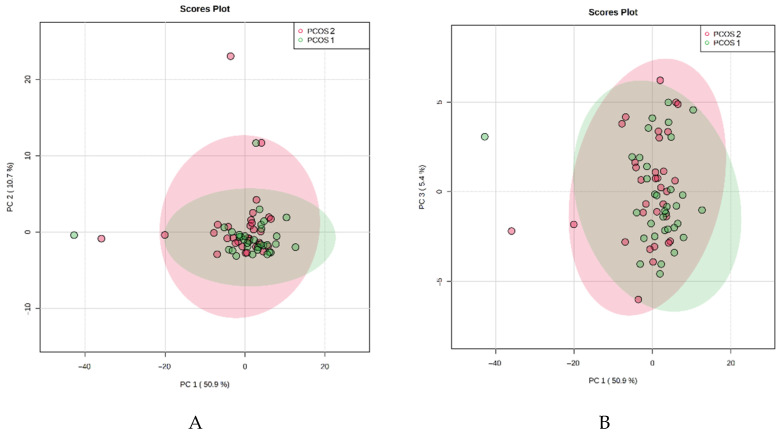
The unsupervised multivariate principal component analysis (PCA) of the studied metabolites in the PCOS-1 and PCOS-2 groups. The plots represent the first principal component (PC1) against the second principal component (PC2) (**A**) and the first principal component (PC1) against the third principal component (PC3) (**B**).

We analyzed PCA results in the next step of the study after dividing them into PCOS-1, PCOS-2, and control groups (Figure 2 and Figure 3).

Similarly, we noticed increased concentration within the PCOS groups compared to healthy controls. In addition, we observed some outliners in both PCOS groups. It seems that not the bodyweight but PCOS itself alternates the metabolic profile of patients. When we analyze the results presented in Figure 1, Figure 2 and Figure 3, we also observe that the control group metabolites have a more visible tendency to concentrate. The PCOS results in both groups seem to spread more intensively and have more outliners. It indicates that the healthy controls have a more constant metabolic profile than all PCOS patients.

### 4.2. Univariate Tests

**Figure 4:**
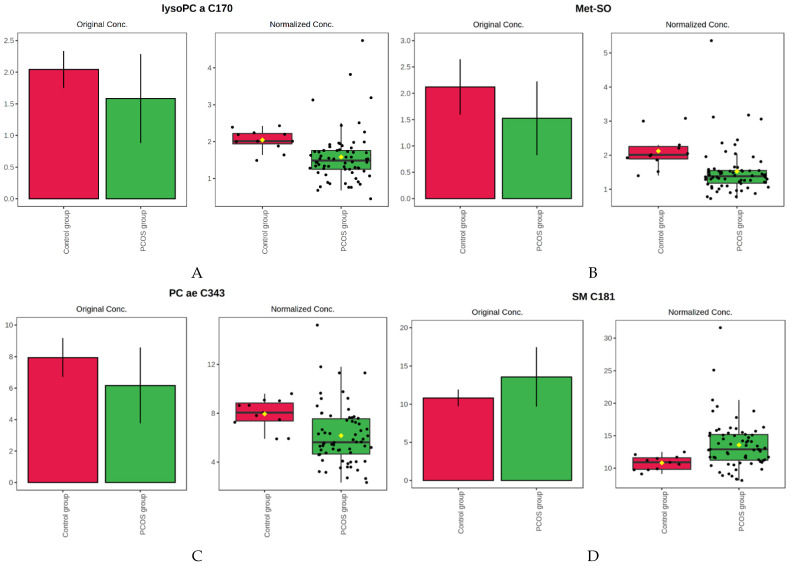
Box plots showing distributions of the selected metabolites across the studied groups. The bar charts on the left show the original values (mean +/− SD). The box and whisker plots on the right summarize the normalized values. However, since these analyzes are univariate, therefore there was no data normalization. Thus, the original values and normalized values make the same values, only shown in two different types of graphs. (**A**) Lysophosphatidylcholine acyl C 17:0 (lyso PC a C17:0); (**B**) Methionine sulfoxide (Met-SO); (**C**) Phosphatidylcholine acyl-alkyl C34:3 (PC ae C34:3); (**D**) Sphingomyelin C18:1 (SM C18:1).

Comparing patients with PCOS and the control group, 30 features from the metabolome dataset had *p* values below 0.05. In the volcano plot, which is the combination of fold change and t-tests, the following variables met the set criteria (fold change threshold 1.25 and the *p*-value threshold 0.05) when comparing the whole PCOS population to the control group: phosphatidylcholine acyl-acyl C36:5 (PC aa C36:5); phosphatidylcholine acyl-alkyl C40:5 (PC ae C40:5); phosphatidylcholine acyl-acyl C38:3 (PC aa C38:3); phosphatidylcholine acyl-alkyl C38:6 (PC ae C38:6); phosphatidylcholine acyl-acyl C36:4 (PC aa C36:4); sphingomyelin C18:1 (SM C18:1) (significantly up-regulated in samples from PCOS patients) and methionine sulfoxide (Met-SO); lysophosphatidylcholine acyl C 18:2 (lysoPC a C18:2); phosphatidylcholine acyl-alkyl C38:2 (PC ae C38:2); lysophosphatidylcholine acyl C17:0 (lysoPC a C17:0); phosphatidylcholine acyl-alkyl C34:3 (PC ae C34:3):, which were significantly down-regulated in PCOS group. In Figure 4, we present four metabolites’ box plots, displaying the most visible difference between the PCOS and the control group.

Those results, as well as univariate ROC curve analysis, are demonstrated in Table 4. We used the ROC curve as an indicator of the highest discriminating potential, which we observed in Methionine sulfoxide (Met-SO) AUC (95%CL) 0.82989 and Lysophosphatidylocholine acyl C 17:0 (lysoPC a C17:0) (AUC (95%CL) 0.8292.

We made a similar comparison between two PCOS groups with 20 metabolites meeting the criteria (fold change threshold 1.25 and the *p*-value threshold 0.05) (Table 5). All the observed metabolites were up-regulated in the PCOS-2. The highest ROC AUC was observed in Phosphatidylcholine acyl-alkyl C34:3 (PC ae C34:3) (AUC 0.788); Phosphatidylcholine acyl-alkyl C40:4 (PC ae C40:4) (AUC 0.778); Phosphatidylcholine acyl-alkyl C40:5 (PC ae C40:5) (AUC 0.785) and Phosphatidylcholine acyl-alkyl C42:5 (PC ae C42:5) (AUC 0.774).

We tried to seek some correlation between metabolome results and clinical parameters characteristic for PCOS patients and those that in many studies differ in PCOS groups compared to healthy controls and metabolome studies. We correlated waist circumference, WHR, fasting insulin, testosterone levels with selected metabolites. We found no significant correlation between those parameters. The strongest positive correlation was R = 0.23 (Lysine and Testosterone), followed by R = 0.225 (acylcarnitine C5 and WHR), and the most apparent negative correlation we found between PC ae C343 and WHR R = (−0.46) and PC ae C343 and waist circumference R = (−0.44).

## 5. Discussion

Metabolomics is an area of science covering comprehensive study and analysis of small-molecule metabolites in various biological systems that create specific organism individual patterns of molecules called metabolites. It is influenced by genetic background, environment, lifestyle, age, and diet, and therefore provides information about the state of a cell and/or organism [43]. The presented study examined women’s metabolome to identify altered metabolite pathways that may provide new insight into PCOS’s underlying biology. The study included patients with average body weight, as well as excess body weight and obesity. Imbalance in body weight and abdominal visceral adiposity is persistent in these women [20,44]. The tendency to generate androgen excess, abdominal adiposity, general obesity, insulin disturbances, and other metabolic syndrome symptoms in PCOS women may be altered already prenatally and in the early stages of life [44].

PCOS includes a lot of abnormalities, which influence several metabolic pathways. It is primarily characterized by disturbed metabolism of the steroid hormones, amino acids, carbohydrates, lipids, purines, and the citric acid cycle [29]. There are not many studies concerning the application of metabolomics in the field of PCOS. Most studies showed that altered metabolites in PCOS were primarily carbohydrate, fat, and protein metabolism [37,45,46,47].

In our study, the main differences between the studied population and controls were in the class of glycerophospholipids, which are one of the major components of cellular membranes, synthesized from glycerol-3-phosphate (G3P) in a de novo pathway that initially produces phosphatidic acid (PA) and diacylglycerol (DAG) or cytidine diphosphate-DAG (CDP-DAG) [48,49,50]. Those de novo pathways generate various types of glycerophospholipids. They differ with polar heads at the *sn*-3 position, like in the glycerol backbone and phosphatidylcholine (PC), phosphatidylethanolamine (PE), phosphatidylserine (PS), phosphatidylinositol (PI), phosphatidylglycerol (PG), and cardiolipin (CL) [51,52], and then further being remodeled in Lands’ cycle [53].

In our PCOS group, up-regulation was observed in lysoPC a C18:2, PC ae C38:2, lysoPC a C17:0, PC ae C34:3. We noticed significant down-regulation of PC aa C36:5, PC aa C40:5, PC aa C40:6, PC aa C38:3, PC aa C38:6, PC aa C36:4. Haoula et al. [54] indicated mainly down-regulation of glycerophospholipids in the PCOS group. The results published by Zhao et al. [45] showed that all of the determined fatty acids are up-regulated in PCOS compared to controls. However, a contrary observation occurs in the case of phosphatidylcholine (PC), phosphatidylethanolamine (PE), and its derivatives lysophosphatidylcholine (LPC) and lysophosphatidylethanolamine (LPE). Those metabolites that include the glycophospholipids mentioned above are mainly decreased in PCOS. We know that lipids are the largest group of molecules and one of the most important ones whose metabolism differs in PCOS [29]. They are responsible for steroid hormone biosynthesis, metabolism of sphingolipids, and fatty acids. We did not find data in previous studies concerning particular metabolites, which were most significantly up-and down-regulated in our research. Nevertheless, previous studies reported decreased levels of similar metabolites: especially LPE (22:5) and LPC, mainly LPC (18:2) [55,56,57]. They take part in glucose metabolism. For example, decreased LPC concentration (18:2) shows the correlation between IR and the risk of developing type 2 diabetes mellitus. These are the disturbances to which women with PCOS seem to be more prone [55].

Nevertheless, similarly to those studies, we could not find a single lipid biomarker or a single pattern of plasma concentrations of lipids that could be characteristic of PCOS. We instead observed that there is a general difference in glycerophospholipids when compared to healthy controls.

Tonks et al., in their lipidomic study, indicated that lower levels of LysoPC correlate with insulin resistance, irrespective of body weight [58]. Interestingly, the whole PCOS group had lower concentrations of lysoPC a C18:2 and lysoPC a C17:0 than controls in our research. When we analyzed those metabolites when PCOS groups were divided according to body weight, women with normal body weight and PCOS had higher concentrations of lysoPC a C18:2, lysoPC a C26:0, lysoPC a C20:4, and lysoPC a C18:1, than overweight and obese women with PCOS. Thus, there was a difference between normal-weight PCOS and controls according to insulin resistance levels in our population, although BMI levels were similar. It seems that insulin resistance and body weight, and PCOS itself, might also impact the levels of LysoPC.

Acylkarnitines are oxidative metabolites, so-called by-products of non-complete fatty acid oxidation [59] built by fatty acid esterified to a carnitine molecule. Mitochondrial and peroxisomal enzymes synthesize them to transport long-chain fatty acids across the mitochondrial membrane for β-oxidation [60,61]. Dysregulation of fatty acid oxidation (FAO), known as lipotoxicity, is recognized as necessary in the pathophysiology of obesity, insulin resistance, and type 2 diabetes [62].

Muoio et al. proposed that acylcarnitines may play an alternative role in the induction of insulin resistance in glucose and lipid metabolism. They described a mechanism in which FAO rate outpaces the tricarboxylic acid cycle (TCA), which causes the accumulation of acylcarnitines, which may impact insulin sensitivity [63,64]. Acylcarnitines are formed mainly from FAO, but they may also be derived from almost any CoA ester [65], ketone bodies [66] degradation products of lysine, tryptophan, valine, leucine, and isoleucine, and carbon atoms from glucose (acetylcarnitine) [65]. From a physiological view, diets and fasting modulate the plasma acylcarnitine profile [67]. Michalik et al. stated that individual long-chain acylcarnitines were increased in obese and type 2 diabetes mellitus (T2DM) patients relative to lean controls. When they compared T2DM patients with thin and obese controls, diabetic patients had significantly elevated several levels. Additionally, in the group with diabetes, those with higher BMI had significant elevations of C_4_– and C_6_–CN relative to lean subjects, although the mean values for C_4_–CN were nearly identical in obese controls and T2DM [62].

Interestingly, in our study, both the control group, which mainly consisted of lean women and normal-weight PCOS women, had significantly higher acylcarnitine C2 than obese women with PCOS. Furthermore, Newbern et al. reported that C2 acylcarnitine was divided by the sum of C3 and C5, inversely correlated with HOMA-IR [68], which would be in line with our observations that the highest concentrations of C2 were in the control group, with the lowest HOMA-IR.

We also observed a significant difference in the concentration of Sphingomyelin C18:1 between the PCOS group and controls. Sphingomyelins are a part of an involved family of sphingolipids, which take part in various biological processes such as cell proliferation, differentiation, apoptosis, migration; membrane trafficking; cell–cell interactions; and cell morphology, as well as both intracellular and extracellular signaling [69,70]. Bikmann et al. indicated that changes in the levels of sphingolipids directly affect the intensity of insulin signaling; depending on the type of sphingolipid, they either promote insulin resistance or enhance the insulin signaling and thus inhibit insulin resistance [71]. Hanamatsu et al. suggested that SM with long saturated acyl chains (18:0, 20:0, 22:0, and 24:0) are involved in developing the metabolic syndrome and its indices such as abnormal lipid profile, obesity, and IR [72]. Li et al. and Haoula et al. observed notable elevation in the sphingomyelin levels in PCOS patients. In the presented study, we noticed a significant increase in the concentration of Sphingomyelin C18:1 in all the patients with PCOS in comparison to Controls. Thus, SM seems to be involved in the pathogenesis of PCOS [73]. BMI does not seem to have an impact on the concentration of SM.

Our study observed significant differences in glycerophospholipid concentrations between the PCOS and the control group. Still, there is no one specific pattern of either up or down-regulation of those compounds.

These observations that present the metabolomic changes between obese and lean women with PCOS for the first time are the most important findings of our study and might indicate new directions for further research on the PCOS population.

## 6. Conclusions

- Our study observed the main differences in glycerophospholipid concentrations but no specific pattern of either up or down-regulation of those compounds.
- We see a tendency to different glycerophospholipids concentrations in PCOS than in a healthy population than a general elevation or decrease.
- Metabolic changes such as insulin resistance and elevated body weight seem to influence acylcarnitine C2 levels more significantly than PCOS itself.
- SM C18:1 should be more intensively observed and examined in future studies and may serve as one of the PCOS biomarkers.

## Figures and Tables

**Table 1:** Characteristic and comparison of whole PCOS group and controls.

	Median (25th–75th Percentile)		
	PCOS (*n* = 66)	Control Group (*n* = 11)	*p*-Value *
Age (years)	28.5 (28.0–32.0)	26 (24.7–30.5)	0.56
BMI (kg/m^2^)	27.4 (22.5–32.9)	21.6 (19.4–41.5)	0.02
Systolic BP (mmHg)	111.0 (100.0–120.0)	108 (100–116)	0.4
Diastolic BP (mmHg)	70.0 (60.0–80.0)	70 (65–71)	0.6
WC (cm)	88.6 (75.0–103.4)	71.6 (66.5–74.8)	0.001
HC (cm)	107.0 (95.3–116.8)	95.4 (91.2–99.8)	0.01
WHR	0.8 (0.8–0.9)	0.75 (0.71–0.8)	0.0005
Fasting insulin (μIU/mL)	11.6 (8.6–17.9)	6.5 (4.5–8.2)	0.0003
Fasting glucose (mg/dL)	92.7 (87.0–97.0)	81.8 (74.3–90)	0.001
HOMA-IR	2.7 (2.0–4.2)	1.3 (0.9–1.6)	0.0001
HBA1%C	5.2 (4.9–5.4)	5.0 (4.8–5.1)	0.1
TC (mg/dL)	172.4 (154.0–196.4)	153.9 (143.3–166.8)	0.02
HDL (mg/dL)	58.9 (49.1–72.1)	70.8 (62.0–80.5)	0.04
LDL (mg/dL)	92.5 (73.0–113.4)	71.4 (61.7–81.9)	0.003
TG (mg/dL)	92.2 (69.8–147.2)	58.3 (47.6–58.8)	0.0006
CRP (mg/L)	0.89 (0.33–2.9)	0.8 (0.2–1.3)	0.2
D-DIMER (ng/mL)	190.0 (109.0–299.0)	254.3 (209.0–303.0)	0.2
Fibrinogen (g/L)	2.9 (2.5–3.6)	2.8 (2.4–3.2)	0.4

* Mann-Whitney, BMI—body mass index, BP—blood pressure, WC—waist circumference, HC—hip circumference, WHR—waist-to-hip ratio, HOMA-IR—Homeostatic Model Assessment for Insulin Resistance, HbA1%C—glycosylated hemoglobin, TC—total cholesterol, HDL—high-density lipoprotein cholesterol, LDL—low-density-lipoprotein cholesterol, TG—triglycerides, CRP—c-reactive protein.

**Table 2:** Characteristic and comparison PCOS-1 vs. PCOS-2 vs. controls.

		Median (25th–75th Percentile)			
Variable	PCOS-1 (*n* = 34) [1]	PCOS-2 (*n* = 32) [2]	Control (*n* = 11) [3]	*p*-Value *	*p*-Value ^#^
Age (years)	28.5 (27–30)	30.8 (28.0–34.0)	26 (24.7–30.5)	0.6	
BMI (kg/m^2^)	32.2 (28.7–35.8)	24.0 (20.7–24.8)	21.6 (19.4–41.5)	<0.0001	1 vs. 3 <0.0001 1 vs. 2 <0.0001 2 vs. 3 1.0
Systolic BP (mmHg)	115 (90–150)	110.1 (100–120.0)	108 (100–116)	0.5	
Diastolic BP (mmHg)	70 (60–100)	70.6 (60.0–80.0)	70 (65–71)	0.8	
WC (cm)	102.6 (82.6–147.5)	74.3 (67.0–80.2)	71.6 (66.5–74.8)	<0.0001	1 vs. 3 <0.0001 1 vs. 2 <0.0001 2 vs. 3 1.0
HC (cm)	116 (105.2–144)	92.9 (88.0–98.0)	95.4 (91.2–99.8)	<0.0001	1 vs. 3 <0.0001 1 vs. 2 <0.0001 2 vs. 3 1.0
WHR	0.88 (0.72–1.17)	0.8 (0.75–0.84)	0.75 (0.71–0.8)	<0.0001	1 vs. 3 <0.0001 1 vs. 2 <0.0001 2 vs. 3 1.0
Fasting insulin (μIU/mL)	16.7 (4.5–66.3)	10.0 (6.7–12.0)	6.5 (4.5–8.2)	<0.0001	1 vs.3 0.0001 1 vs. 2 0.007 2 vs. 3 <0.0001
Fasting glucose (mg/dL)	92.7 (85.7–97.0)	91.1 (87.6–96.4)	81.8 (74.3–90)	0.005	1 vs. 3 0.86 1 vs. 2 1.0 2 vs. 3 0.8
HOMA-IR	4.6 (2.7–5.6)	2.3 (1.5–2.7)	1.3 (0.9–1.6)	<0.0001	1 vs. 3 <0.0001 1 vs. 2 0.003 2 vs. 3 <0.0001
HBA1%C	5.4 (5.1–5.6)	5.0 (4.8–5.2)	5.0 (4.8–5.1)	0.003	1 vs. 3 <0.0001 1 vs. 2 <0.0001 2 vs. 3 1.0
TC (mg/dL)	181.1 (163.6–191.1)	177.2 (144.8–200.4)	153.9 (143.3–166.8)	0.03	1 vs. 2 0.097 1 vs. 3 0.001 2 vs. 3 0.002
HDL (mg/dL)	50.2 (38.9–56.5)	72.9 (59.2–83.1)	70.8 (62.0–80.5)	<0.0001	1 vs. 2 <0.0001 1 vs. 3 <0.0001 2 vs.3 1.0
LDL (mg/dL)	106.9 (83.3–125.9)	86.8 (60.8–106.9)	71.4 (61.7–81.9)	0.0002	1 vs.2 0.03 1 vs. 3 0.001 2 vs.3 0.4
TG (mg/dL)	151.9 (90.8–200.5)	87.4 (55.6–116.7)	58.3 (47.6–58.8)	<0.0001	1 vs. 2 0.001 1 vs. 3 <0.0001 2 vs. 3 0.04
CRP (mg/L)	2.9 (0.8–4.2)	0.8 (0.12–0.9)	0.8 (0.2–1.3)	0.0003	1 vs. 2 0.003 1 vs. 3 0.003 2 vs. 3 1.0
D-DIMER (ng/mL)	244.3 (113.0–327.0)	196.2 (96.0–290.0)	254.3 (209.0–303.0)	0.23	
Fibrinogen (g/L)	3.4 (2.8–3.8)	2.7 (2.27–2.9)	2.8 (2.4–3.2)	0.004	1 vs. 2 0.01 1 vs. 3 1.0 2 vs. 3 0.01

* Kruskal-Wallis, ^#^ Bonferroni correction test, BMI—body mass index, BP—blood pressure, WC—waist circumference, HC—hip circumference, WHR—waist-to-hip ratio, HOMA-IR—Homeostatic Model Assessment for Insulin Resistance, HbA1%C—glycosylated hemoglobin, TC—total cholesterol, HDL—high-density lipoprotein cholesterol, LDL—low-density-lipoprotein cholesterol, TG—triglycerides, CRP—c-reactive protein.

**Table 3:** Hormonal analysis: PCOS-1 vs. PCOS-2 vs. controls.

	Median (25th–75th Percentile)			
Variable	PCOS-1 (*n* = 34)	PCOS-2 (*n* = 32)	Control (*n* = 11)	*p*-Value *
FSH (mIU/mL)	6.0 (4.7–6.7)	6.4 (5.6–7.6)	5.1 (4.0–6.3)	0.08
LH (mIU/mL)	11.8 (7.8–14.4)	15.6 (11.2–19.0)	6.0 (5.0–7.1)	*p* < 0.001
E2 (pg/mL)	75.6 (43.2–60.8)	66.3 (45.2–76.8)	88.0 (68.0–99.0)	0.75
PRL (ng/mL)	13.0 (8.4–15.5)	12.3 (7.7–13.0)	13.8 (6.8–16.4)	0.78
T (ng/mL)	0.7 (0.4–0.8)	0.54 (0.42–0.63)	0.43 (0.3–4.8)	0.03
DHEA-S (ug/dL)	277.0 (202.0–350.0	237.0 (198.0–322.0)	140.0 (122.0–171.0	0.04
TSH (µU/mL)	1.9 (1.3–2.5)	1.8 (1.2–2.4)	1.6 (1.2–2.1)	0.85
SHBG (nmol/L)	31.1 (18.2–48.4)	45.5 (42.8–47.6)	56.0 (41.1–68.4	*p* < 0.001
17-OH-P (ng/mL)	0.9 (0.7–1.0)	0.8 (0.6–0.9)	0.7 (0.6–0.8)	0.8

* Values are expressed as medians (interquartile range); *p*-values were calculated using the Mann-Whitney post hoc U-test with Bonferroni adjustment; *p* < 0.05 was considered statistically significant. DHEA-S—dehydroepiandrosterone sulfate; E2—estradiol; FSH—follicle-stimulating hormone; PRL—prolactin; SHBG—sex-hormone-binding globulin; T—testosterone; TSH—thyroid-stimulating hormone; 17-OH-P—17-hydroxy-progesterone.

**Table 4:** List of differentiating metabolites with their serum concentrations determined in the whole PCOS group and control group (mean ± SD, uM) and results from univariate statistics.

Metabolite	Abbreviation	PCOS Group (Serum Concentration)	Control Group (Serum Concentration)	*p*-Value	Fold Change	AUC (95%CI)
Phosphatidylocholine acyl-acyl C36:5	PC aa C36:5	21.59 ± 11.27	14.45 ± 3.70	0.0131	0.66901	0.73554
Methionine sulfoxide	Met-SO	1.52 ± 0.70	2.12 ± 0.53	0.0005	1.3909	0.82989
Lysophosphatidylcholine acyl C 18:2	lysoPC a C18:2	22.71 ± 10.28	31.48 ± 9.11	0.0074	1.3861	0.75413
Phosphatidylcholine acyl-alkyl C38:2	PC ae C38:2	1.37 ± 0.67	1.82 ± 0.67	0.0187	1.3305	0.72314
Phosphatidylocholine acyl-acyl C40:5	PC aa C40:5	8.99 ± 3.74	6.87 ± 1.29	0.0423	0.76456	0.69284
Lysophosphatidylcholine acyl C 17:0	lysoPC a C17:0	1.58 ± 0.70	2.04 ± 0.29	0.0005	1.2908	0.8292
Phosphatidylcholine acyl-acyl C38:3	PC aa C38:3	47.16 ± 16.38	36.56 ± 7.74	0.0173	0.7753	0.7259
Phosphatidylcholine acyl-alkyl C34:3	PC ae C34:3	6.17 ± 2.41	7.95 ± 1.23	0.0024	1.2882	0.78788
Phosphatidylocholine acyl-acyl C38:6	PC aa C38:6	72.64 ± 26.86	57.37 ± 14.52	0.0336	0.78983	0.70179
Phosphatidylcholine acyl-acyl C36:4	PC aa C36:4	157.89 ± 47.26	125.18 ± 17.19	0.0058	0.79283	0.76171
Sphingomyelin C18:1	SM C18:1	13.57 ± 3.88	10.82 ± 1.10	0.0044	0.79725	0.76977

**Table 5:** Differentiating metabolites with serum concentrations determined between PCOS-1 and PCOS-2 (mean ± SD, uM) and results from univariate statistics.

Metabolite	Abbreviation	PCOS-1 (Serum Concentration)	PCOS-2 (Serum Concentration)	Fold Change	AUC (95%CI)	*p*
Lysophosphatidylcholine acyl C 18:2	lysoPC a C18:2	17.72 ± 5.98	22.47 ± 9.35	1.3861	0.75413	0.001
Phosphatidylcholine acyl-alkyl C34:3	PC ae C34:3	5.31 ± 2.04	7.07 ± 2.48	1.2882	0.78788	0.0001
Phosphatidylcholine acyl-alkyl C44:5	PC ae C44:5	1.72 ± 0.65	2.26 ± 0.81	1.3119	0.749	0.0001
Phosphatidylcholine acyl-alkyl C42:1	PC ae C42:1	0.22 ± 0.07	0.28 ± 0.12	1.3037	0.73024	0.0003
Phosphatidylcholine acyl-alkyl C40:3	PC ae C40:3	1.12 ± 0.65	1.45 ± 1.34	1.3026	0.626	0.066
Phosphatidylcholine acyl-alkyl C40:4	PC ae C40:4	1.91 ± 0.70	2.48 ± 0.93	1.2974	0.77574	0.00001
Sphingomyelin C22:3	SM C22:3	1.44 ± 0.62	1.85 ± 0.63	1.2919	0.713	0.0012
Phosphatidylcholine acyl-alkyl C34:2	PC ae C34:2	7.56 ± 3.03	9.74 ± 3.81	1.2885	0.71	0.013
Phosphatidylcholine acyl-alkyl C44:3	PC ae C44:3	0.09 ± 0.04	0.12 ± 0.05	1.277	0.71	0.0008
Phosphatidylcholine acyl-alkyl C40:5	PC ae C40:5	3.04 ± 1.46	3.88 ± 1.62	1.2768	0.785	0.00001
Phosphatidylcholine acyl-alkyl C42:4	PC ae C42:4	0.77 ± 0.28	0.98 ± 0.32	1.2758	0.76057	0.00001
Phosphatidylcholine acyl-alkyl C44:6	PC ae C44:6	1.06 ± 0.40	1.34 ± 0.46	1.2701	0.7284	0.0002
Phosphatidylcholine acyl-alkyl C38:2	PC ae C38:2	1.21 ± 0.55	1.53 ± 0.75	1.3305	0.688	0.0046
Lysophosphatidylcholine acyl C 18:1	lysoPC a C18:1	17.72 ± 5.98	22.47 ± 9.35	1.2681	0.664	0.015
Acylkarnitine C2	C2	5.96 ± 2.26	7.49 ± 4.09	1.2603	0.571	0.336
Phosphatidylcholine acyl-alkyl C42:5	PC ae C42:5	1.99 ± 0.65	2.50 ± 0.68	1.2589	0.7739	0.00001
Lysophosphatidylcholine acyl C 26:0	lysoPC a C26:0	0.11 ± 0.03	0.13 ± 0.07	1.2547	0.659	0.02
Phosphatidylcholine acyl-alkyl C40:1	PC ae C40:1	0.87 ± 0.30	1.09 ± 0.39	1.2514	0.689	0.002
Phosphatidylcholine acyl-alkyl C38:3	PC ae C38:3	3.68 ± 1.57	4.60 ± 2.93	1.2511	0.601	0.15
Lysophosphatidylcholine acyl C 24:0	lysoPC a C20:4	5.79 ± 1.90	7.24 ± 2.62	1.2507	0.693	0.00028

## Data Availability

Not applicable.
